# Is detrusor underactivity the urodynamic characteristic of long-COVID in patients with benign prostate hyperplasia?

**DOI:** 10.1097/MD.0000000000040156

**Published:** 2024-10-11

**Authors:** Ning Xiao, Jinhua Xiao, Qi Tang, Gaoyu Pan, Kailu Wei, Huasheng Zhao, Jianfeng Wang

**Affiliations:** a Department of Urology, Video Urodynamic Studies Center, The Second Affiliated Hospital of Guilin Medical University, Guilin, China; b Department of Urology, Continence Research Clinic, Shaoyang Central Hospital, Shaoyang, China; c Department of Urology, Shaoyang Hosptial Affiliated to University of South China, Shaoyang, China; d Department of Trauma, The Second Affiliated Hospital of Guilin Medical University, Guilin, China.

**Keywords:** benign prostate hyperplasia, bladder outlet obstruction, COVID-19, detrusor underactivity, lower urinary tract symptoms, urodynamic studies

## Abstract

Although coronavirus disease 19 (COVID-19) was reported to involve with multiple organs, COVID-19 reports focusing on urinary system mostly evaluated the association between lower urinary tract symptoms and COVID-19 using questionnaire score. In this study, sonography video urodynamic studies was first conducted to explore the effects of COVID-19 on contractility of bladder detrusor of patients with benign prostate hyperplasia (BPH). Clinical data was respectively reviewed and compared between BPH patients with previous COVID-19 infection (COVID-19 group) and without previous COVID-19 (non-COVID-19 group). The incidence of detrusor underactivity (DU) was compared between 2 groups. Comparison of age and noninvasive parameters was conducted between BPH patients with DU and without DU in COVID-19 group. Correlation coefficient between noninvasive parameters and detrusor contractility was determined and receiver operating characteristic curve of noninvasive parameters was used to choose the most appropriate cutoff for detection of DU in COVID-19 group. Beside a significant increase in the incidence of DU in BPH patients of COVID-19 group, a lower detrusor contractility and a greater bladder wall thick was detected compared to that of patients in non-COVID-19 group. Post-voiding residual urine was found to have a linear correlation with detrusor contractility in COVID-19 group. It was suggested that COVID-19 infection would further exacerbate impairment of detrusor previously resulted from bladder outlet obstruction in BPH patients. DU may be a urodynamic characteristic of long-COVID.

## 1. Introduction

Since December 2019, the acute respiratory disease, known as coronavirus disease 19 (COVID-19) has become a pandemic affecting a significant number of people in the world and resulting in irreversible complications, which involved with multiple organs, such as respiratory, bone marrow, and cardiovascular system.^[1]^ In the past no less 4 years since outbreak of COVID-19, there have been reports that de novo or aggravated lower urinary tract symptoms (LUTS) is associated with infection of COVID-19 and a higher score of LUTS in patients with COVID-19 is an indicator of poor prognosis.^[2,3]^

However, there has been some controversies over relationship between COVID-19 and LUTS due to possibly heterogeneous recruited patients in different reports.^[2,4,5]^ Exacerbation of LUTS after COVID-19 infection, including increased international prostate symptoms score and quality of life, has been discerned in elderly male.^[6]^ Moreover, there was reported to be a higher incidence of urinary retention in benign prostatic hyperplasia (BPH) patients with COVID-19 compared to that of without.^[7]^ Given the expression of angiotensin converting enzyme 2 (ACE2) receptor in urogenital system, some hypotheses, including COVID-related cystitis, inflammatory storm, and psychological stress, have been considered to play a key role in exacerbation of LUTS in patients with COVID-19, but which has been not proven by animal studies.^[8–10]^

In men over age 50, BPH is the most common male urological disease and aggravation of LUTS due to progression of the disorder was not uncommon in patients with BPH ultimately requiring surgical interventions.^[11]^ Almost all previously studies, focusing on the associations between COVID-19 infection and LUTS in BPH patients, adopted the symptoms questionnaire scores, including international prostate symptoms score and quality of life, and ultrasound parameters, such as post-voiding residual urine (PVR) and prostate volume (PV).^[12]^ Given that both bladder outlet obstruction (BOO) and contractility of detrusor can respectively or mutually affect LUST and PVR, questionnaire scores and ultrasound parameters lack the ability to differentiate BOO from detrusor underactivity (DU).^[13]^

Since increased LUTS of elderly male during COVID-19 treatment and poor prognosis in BPH patients with more severe LUTS, here urgently needs an insight into relationship between LUTS and function of bladder detrusor in BPH patients with COVID-19 infection.^[3,14]^ To the best of our knowledge, except for a case report, there has not been an observational study adopting invasive urodynamic studies (UDS) in evaluation of lower urinary tract function in COVID-19 patients.^[15]^ Therefore, the contractility of detrusor in BPH patients with COVID-19 was first urodynamically compared with that of without in this study.

## 2. Materials and methods

The clinical datasets of BPH patients underwent pressure-flow study of UDS, who presenting intractable LUTS for BPH or other conditions requiring prostate de-obstruction interventions, between January 2023 and June 2023 were reviewed at the Second Affiliated Hospital of Guilin Medical University and Shaoyang Central Hospital. Those patients were divided into 2 groups, in which COVID-19 group was composed of patients previously diagnosed with COVID-19 infection, who were confirmed with the real-time reverse transcriptase-polymerase chain reaction of oropharyngeal and nasopharyngeal swabs obtained as per the World Health Organization guidelines between 2 and 4 weeks prior to UDS, and patients without previous COVID-19 infection were regarded as non-COVID-19 group. Negative results were required in consecutive 2 times real-time reverse transcriptase-polymerase chain reaction for COVID-19, the interval of which was between 24 hours and 48 hours, before UDS in enrolled patients in this study following local authorities guidelines. Exclusion criteria in the present study included voiding volume <150 mL, previous prostatic and urethral surgery, urethral stenosis, neurogenic bladder, utilization of medication that affects micturition, proven prostate or bladder carcinoma, urinary infection, and pelvic radiotherapy. This study was in accord with the ethical principles of the Declaration of Helsinki and informed consents were obtained from all subjects.

The sonography video SVUDS combined an ultrasound scan with multichannel UDS (Aquarius XT, Laborie, USA) and could synchronously integrate urodynamic measurement values with sonographic images sequences by Aquarius XT own software (UDS.V14, Laborie, USA). PV, intravesical prostate protrusion (IPP), and bladder wall thick (BWT) were measured by a single urologist through transabdominal ultrasound scan (DC-65, Mindray, China) at a bladder volume of 150 to 200 mL using 7.5-MHz line array during SVUDS according to a previous report.^[16]^ Peak flow rate (Qmax) was measured by uroflowmetry and PVR was determined by ultrasound scan. Maximum detrusor pressure during pressure-flow study (Pdet.max), detrusor pressure at Qmax (Pdet.Qmax), BOO index (BOOI; calculated as Pdet.Qmax - 2Qmax) and bladder contractility index (BCI; calculated as Pdet.Qmax + 5Qmax) were determined during SVUDS. Maximum Watts factor (WFmax) and Chess classification, including footpoint and curvature, were automatically generated by UDS.V14. Although there have been controversies over the numerical value of WFmax in definition of DU, a BCI of <100 has been considered DU.^[17]^ All SVUDS were underwent by a urologist in according with the Good urodynamic Practices of International Continence Society.^[18]^

*T* test, if data were normally distributed, or the Mann–Whitney *U* test, in case variables were nonnormal distribution, was conducted to determine the differences in age, PV, IPP, PVR, BWT, Qmax, Pdet.max, Pdet.Qmax, BOOI, BCI, WFmax, footpoint, and curvature between 2 groups. The chi-square test was used to determine whether there was a difference in the incidence of DU between 2 group. *T* test or Mann–Whitney *U* test was adopted to detect the differences of noninvasive parameters, including age, PV, IPP, PVR, BWT, and Qmax, between BPH patients with DU and without DU in COVID-19 group. Pearson correlation coefficient when data was normal distribution or Spearman rank correlation coefficient in case variables had a distribution far from normal was performed to assess respectively the relationship between noninvasive parameters and detrusor contractility represented by BCI or WFmax, in BPH patients with COVID-19 infection. Receiver operating characteristic (ROC) curve of noninvasive parameters was used to choose the most appropriate cutoff for detection of DU in COVID-19 group. All statistical analyses were performed using SPSS for Windows (version 27.0, Statistical Package for Social Science, Chicago, IL). A *P* value of <.05 was considered statistically significant.

## 3. Results

One hundred six BPH patients were enrolled in this study, in which previous COVID-19 infection was confirmed in 36 and 70 were recruited into non-COVID-19 group. There was not any significant differences in age (*P* = .457), PV (*P* = .28), IPP (*P* = .712), PVR (*P* = .133), Qmax (*P* = .701), BOOI (*P* = .054), footpoint (*P* = .467), and curvature (*P* = .928) between 2 groups, but, besides greater BWT (*P* = .009), a lower value of Pdet.max (*P* = .023), Pdet.Qmax (*P* = .017), BCI (*P* = .011), and WFmax (*P* = .011) also were found in BPH patients with COVID-19 when compared with that of non-COVID-19 (Table 1).

**Table 1:** Comparison of clinical data between BPH patients with COVID-19 and non-COVID-19.

	COVID-19 (n = 36)	Non-COVID-19 (n = 70)	T/Z	*P* value
Age (year)	69.4 ± 10.2	70.8 ± 9.0	0.746	.457
PV (mL)	44.5 (30.5, 54.5)	47.0 (31.8, 72.3)	−1.081	.28
IPP (cm)	1.5 (1.0, 2.0)	2.0 (1.0, 2.0)	−0.370	.712
PVR (mL)	95.0 (52.5, 137.5)	70.0 (10.0, 140.5)	−1.503	.133
BWT (mm)	2.9 (2.4, 3.7)	2.6 (2.4, 3.0)	−2.601	.009
Qmax (mL/sec)	5.5 (4.5, 9.3)	6.5 (4.7, 8.7)	−0.384	.701
Pdet.max	56.4 (38.6, 88.5)	75.8 (53.9, 90.4)	−2.282	.023
Pdet.Qmax	50.7 ± 20.7	61.1 ± 21.0	2.415	.017
BCI	83.9 ± 21.0	96.6 ± 24.9	2.599	.011
WFmax	8.6 (6.4, 10.1)	10.0 (7.6, 12.2)	−2.542	.011
BOOI	37.4 ± 23.6	46.9 ± 24.0	1.949	.054
Footpoint	26.1 (15.0, 46.1)	34.3 (19.0, 47.5)	−0.727	.467
Curvature	0.6 (0.2, 1.6)	0.6 (0.3, 1.4)	−0.0090	.928

BCI = bladder contractility index, BOOI = BOO index, BWT = bladder wall thick, COVID-19 = coronavirus disease 19, IPP = intravesical prostate protrusion, Pdet.max = maximum detrusor pressure during pressure-flow study, Pdet.Qmax = detrusor pressure at Qmax, PV = prostate volume, PVR = post-voiding residual urine, Qmax = peak flow rate, WFmax = maximum Watts factor.

A higher incidence (75.0%, 27/36) of DU was discerned in BPH patients with COVID-19 when compared with that (54.3%, 38/70) of non-COVID-19 (*P* = .038) (Table 2). Moreover, BPH patients with DU in COVID-19 group had a larger PVR compared to that of normal detrusor contractility (non-DU) (*P* = .048), but age (*P* = .299), PV (*P* = .335), IPP (*P* = .558), BWT (0.441), and Qmax (0.156) did not present any differences (Table 3). In noninvasive parameters, only PVR significantly respectively correlated with BCI (*r* = −0.360, *P* = .031) and WFmax (*r* = −0.508, *P* = .002) of BPH patients with COVID-19 (Table 4).

**Table 2:** The chi-square test for incidence of DU between BPH patients with COVID-19 and non-COVID-19.

	COVID	Non-COVID-19	*χ* ^2^	*P* value
Non-DU	9	32	4.301	.038
DU	27	38		

COVID-19 = coronavirus disease 19, DU = detrusor underactivity.

**Table 3:** Comparison of noninvasive parameters between BPH patients with DU and non-DU in COVID-19 group.

	DU (n = 27)	Non-DU (n = 9)	T/Z	*P* value
Age (year)	70.5 ± 10.1	66.3 ± 10.5	−1.054	.299
PV (mL)	43.9 ± 21.1	51.4 ± 16.4	0.979	.335
IPP (cm)	2.0 (1.0, 2.0)	1.0 (0.5, 2.0)	−0.585	.558
PVR (mL)	110 (70, 140)	60 (10, 90)	−1.976	.048
BWT (mm)	2.9 (2.5, 3.7)	2.8 (2.3, 4.3)	−0.770	.441
Qmax (mL/sec)	6.2 ± 2.7	7.8 ± 3.8	1.449	.156

BWT= bladder wall thick, COVID-19 = coronavirus disease 19, DU = detrusor underactivity, IPP = intravesical prostate protrusion, PV = prostate volume, PVR= post-voiding residual urine, Qmax= peak flow rate.

**Table 4:** The correlation coefficients between noninvasive parameters and BCI and WFmax in BPH Patients with COVID-19.

	Age	PV	IPP	BWT	PVR	Qmax
BCI	−0.232	0.271	0.017	−0.103	−0.360*	0.286
WFmax	−0.166	0.265	−0.108	−0.3	−0.508**	0.207

BCI = bladder contractility index, BWT = bladder wall thick, COVID-19 = coronavirus disease 19, IPP = intravesical prostate protrusion, PV = prostate volume, PVR = post-voiding residual urine, Qmax = peak flow rate, WFmax = maximum Watts factor.

* *P* = .031.

** *P* = .002.

**Figure 1.**
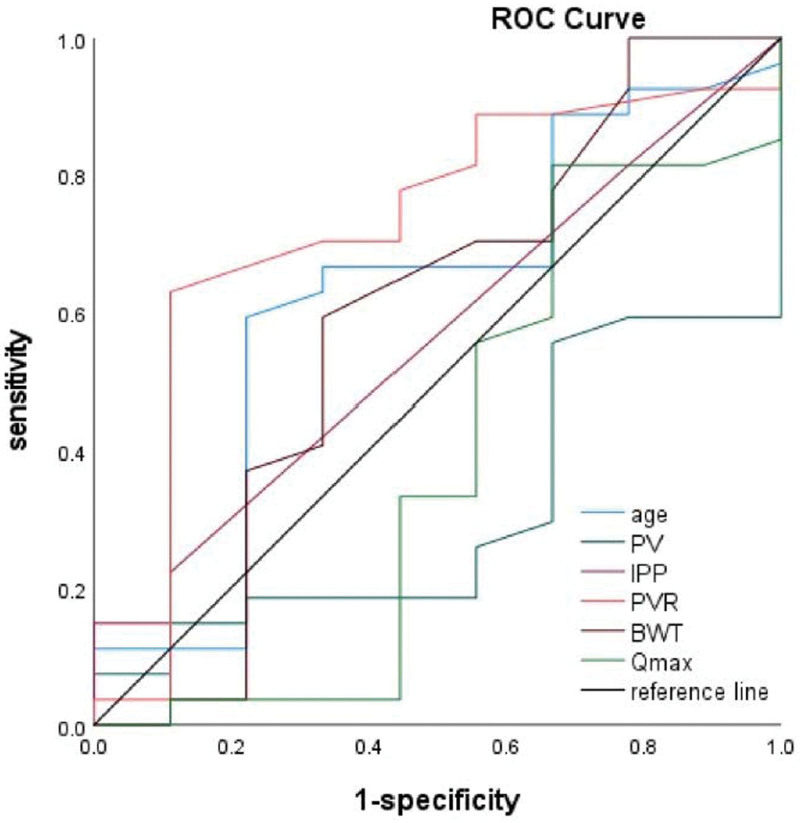
ROC of noninvasive parameters for differentiation of DU in BPH patients with COVID-19. BWT = bladder wall thick, COVID-19 = coronavirus disease 19, DU = detrusor underactivity, IPP = intravesical prostate protrusion, PV = prostate volume, PVR = post-voiding residual urine, Qmax = peak flow rate, ROC = receiver operating characteristic.

In this study, ROC curve was used to clarify the ability of noninvasive parameters in differentiation between DU and non-DU in BPH patients with COVID-19. The results showed that only PVR might have a potential capacity mentioned above with 0.722 of area under curve (AUC) (*P* = .049) and 95 mL of cutoff value of PVR with 0.519 of Youden index presenting 63% of sensitivity and 88.9% of specificity (Table 5 and Fig. 1).

**Table 5:** AUC of ROC of noninvasive parameters for differentiation of DU in BPH patients with COVID-19.

	AUC	SE	*P* value	Asymptotic 95% confidence interval	
				Lower bound	Upper bound
Age	0.621	0.114	.281	0.398	0.845
PV	0.313	0.094	.096	0.129	0.496
IPP	0.564	0.107	.571	0.355	0.773
PVR	0.722	0.104	.049	0.519	0.925
BWT	0.586	0.126	.443	0.339	0.833
Qmax	0.387	0.126	.315	0.141	0.633

BWT = bladder wall thick, COVID-19 = coronavirus disease 19, DU = detrusor underactivity, IPP = intravesical prostate protrusion, PV = prostate volume, PVR = post-voiding residual urine, Qmax = peak flow rate, ROC = receiver operating characteristic.

## 4. Discussions

Various etiological factors have been reported to associate with LUTS in elderly male, in which BPH is the most common one.^[19]^ LUTS was composed of symptoms in storage phase, micturition phase, and post-voiding phase, and storage symptoms, especially urinary frequency, has been more frequently reported when compared with micturition or post-voiding during COVID-19 pandemic.^[5]^ Given that components of renin-angiotensin system were also localized in the prostate gland and renin-angiotensin system dysregulation could play a role in progression of BPH, COVID-19 infection has been considered an etiological cause in LUTS exacerbation of BPH patients derived from exacerbation of inflammation in prostate gland of BPH due to binding of ACE2 receptor in prostate with virus of COVID-19.^[20]^ More severe BOO was supposed to be detected in BPH patients with COVID-19 compared to that of without if the hypothesis mentioned above was true. However, there was not any significant differences of BOOI, footpoint, and curvature between COVID-19 and non-COVID-19 group in this study. Although our findings in this study could not robustly infer the exclusion of the role of BOO progression resulted from inflammation of prostate due to COVID-19 in LUST exacerbation of BPH patients, it was suggested that multiple factors were attributable to deterioration of LUTS.

Beside BOO, bladder detrusor function, including stability and contractility, has been regarded as an important factor impacting LUTS in BPH patients.^[21]^ Over activity of bladder was most often generated from BOO in BPH patients and could be urodynamically proved as detrusor overactivity representing decreased stability of bladder detrusor, due to which patients usually complained frequency, nocturia, and urgency.^[11]^ Thicker detrusor always tried to overcome BOO and was gradually associated with pathological alteration in microstructure of detrusor, for example apoptosis of smooth muscle cells and deposition of collagen fiber, and decompensation of detrusor function and DU would follow in case of failure in effective relief for BOO.^[22]^ BPH patients with BOO and DU would have difficult in voiding, prolonged bladder emptying, and/or failure to completely empty the bladder as well as nocturia, frequency, urgency, urinary retention. Beside BOO, chronic viral or bacterial infection and neurogenic diseases also damaged the function of bladder detrusor. Hence, COVID-associated cystitis (CAC) has been considered the culprit of long-term over activity of bladder following COVID-19 infection but there was not an observational report focusing on contractility of bladder detrusor in COVID-19 patients in the literature, expect for a case report.^[8,15]^

To our surprise, significant lower values of all the indicators of detrusor contractility used in the study, including BCI, WFmax, Pdet.max, and Pdet.Qmax, were found in BPH patients with COVID-19 when compared to that of non-COVID-19. Given that no significant difference in BOO was found between BPH patients with COVID-19 and non-COVID-19 in this study, it was inferred that DU might play a more important role in long-effect of COVID-19 on LUTS of BPH patients compared to congested prostate gland due to COVID-19. Moreover, the incidence of DU was significantly higher (75.0%, 27/36) in BPH patients with COVID-19 when compared to that of non-COVID-19 (54.3%, 38/70) in this study. To the best of our knowledge, a potentially harmful long-effects of COVID-19 on detrusor contractility of BPH patients was first urodynamically found in this study.

However, there has not been a report to conduct animal studies for clarification in the effects of COVID-19 on bladder detrusor across the literature. Given expression of ACE2 receptor on the urothelial cell, it has been hypothesized that direct invasion of COVID-19 into the bladder mucous could cause CAC.^[23]^ Additionally, the finding of elevation of pro-inflammation cytokines, for example IL-6, IL-8, and IP-10, in urine of COVID-19 patients with LUTS and without urinary infection supported another theory that these cytokines secreted in urine could sensitize bladder leading to LUTS.^[24]^

Is only bladder mucous was involved within CAC? However, in present study, detection of thicker BWT, representing inflammation, collagen deposition, and fibrosis proven in animal and human DU studies,^[22]^ and higher incidence of DU in BPH patients with COVID-19 compared to that of non-COVID-19 implied that CAC’s involvement with detrusor may be a contributing factor in LUST of patients with COVID-19. Hence, we hypothesized that inflammation of detrusor attributed to COVID-19 would further exacerbate detrusor damage generated previously by BPH/BOO, due to which DU would become more obvious after COVID-19 infection in BPH patients and might last a long-term, namely “long-COVID” of detrusor. Since no satisfactory results have been obtained for treatment of DU, more researches would be needed to elucidate the mechanisms of DU induced by COVID-19 to prevent timely and effectively detrusor from decompensation.

Although the pressure-flow study of UDS is the “golden standard” for diagnosis of DU, the invasive and expensive nature of UDS required urgently noninvasive methods to replace the UDS.^[25]^ PVR often was investigated using the transabdominal ultrasound to evaluate the differentiation efficiency for DU, but the unreliable performance of PVR has been previously observed in some studies, enrolling non-COVID-19 patients.^[26,27]^ However, in present study, significant larger PVR was determined in BPH patients with DU compared to non-DU and a negative linear correlation was detected between PVR and detrusor contractility, represented by BCI and WFmax, in COVID-19 group. Moreover, PVR was found to have a capacity of differentiation for DU with 0.722 of AUC and 95 mL of cutoff value of PVR with 0.519 of Youden index presenting 63% of sensitivity and 88.9% of specificity. Therefore, we considered PVR as a potentially noninvasive parameter for discerning DU in BPH patients with COVID-19.

Several limitations of the present study should be carefully noted. Firstly, the medical data in this study were obtained for clinical purposes, not for research aim. Secondly, relatively small size and retrospective nature of the present study should be considered. Third, only BPH patients was enrolled into this study. Therefore, more general populations, such as female and male patients without BPH, may facilitate the evaluation of generalizability of our results. Finally, patients were followed up after no more than 1 month and longer following-up was needed in the further research to observe the change of detrusor contractility in BPH patients with DU in COVID-19 group.

## 5. Conclusions

Lower value of BCI, WFmax, Pdet.max, and Pdet.Qmax and thicker BWT observed in BPH patients with previous COVID-19 infection when compared to non-COVID-19 indicated that COVID-19’s involvement with bladder detrusor would cause more poor contractility of detrusor contributing to the increased incidence of DU. Given the long-effects of COVID-19 on detrusor, DU may be a urodynamic characteristic of long-COVID. PVR could be deemed a potentially noninvasive parameter for differentiation of DU in BPH patients with COVID-19. More attentions should pay to preserve timely and effectively the function of detrusor in the era of COVID-19 pandemic.

## Author contributions

**Conceptualization:** Ning Xiao, Jinhua Xiao, Huasheng Zhao.

**Data curation:** Ning Xiao, Jinhua Xiao, Qi Tang, Gaoyu Pan, Kailu Wei, Huasheng Zhao, Jianfeng Wang.

**Formal analysis:** Ning Xiao.

**Funding acquisition:** Ning Xiao.

**Investigation:** Ning Xiao, Qi Tang, Gaoyu Pan, Kailu Wei, Huasheng Zhao, Jianfeng Wang.

**Methodology:** Ning Xiao.

**Project administration:** Ning Xiao.

**Resources:** Ning Xiao.

**Software:** Ning Xiao, Qi Tang.

**Supervision:** Ning Xiao, Jianfeng Wang.

**Validation:** Ning Xiao.

**Visualization:** Ning Xiao, Kailu Wei.

**Writing – original draft:** Ning Xiao, Jinhua Xiao.

**Writing – review & editing:** Ning Xiao.
